# Avian influenza in Ireland: a spatiotemporal, subtype and host-based analysis (1983–2024)

**DOI:** 10.1099/jgv.0.002218

**Published:** 2026-02-25

**Authors:** Maeve Louise Farrell, Guy McGrath, Laura Garza Cuartero, Gerald Barry

**Affiliations:** 1 Veterinary Sciences Centre, School of Veterinary Medicine, University College Dublin, Belfield, Dublin 4, Ireland; 2 University College Dublin One Health Centre, University College Dublin (UCD), Dublin, Ireland; 3 Centre for Veterinary Epidemiology and Risk Analysis (CVERA), University College Dublin (UCD), Dublin, Ireland; 4 Central Veterinary Research Laboratory, DAFM, Backweston Campus, Stacummy Lane, W23 X3PH Celbridge, Ireland

**Keywords:** avian influenza, highly pathogenic avian influenza (HPAI), One Health, spatiotemporal analysis

## Abstract

Avian influenza virus (AIV) is a significant global concern, causing widespread mortality in wild birds, domestic poultry and, more recently, wild and domestic mammals. This study presents a retrospective analysis of AIV detections in the Republic of Ireland. Data were sourced from official surveillance databases, peer-reviewed literature and grey literature sources. Spatio-temporal, host-specific and subtype patterns were assessed using descriptive statistics, chi-square tests, linear regression and kernel density estimations. A total of 2,888 confirmed AIV detections were recorded across 25 of Ireland’s 26 counties. Wild birds accounted for 98.7% of detections, with domestic birds comprising 1.3%, and two detections in foxes. H5N1 highly pathogenic avian influenza (HPAI) was the most prevalent subtype (96.7%), followed by H5N8 HPAI and H6N1. Spatial clustering was observed in urban areas, particularly Dublin. The highest seasonal peak occurred during summer, contrasting with traditional winter-associated patterns. Several detections occurred in migratory species outside of typical residency periods, suggesting potential climate-related shifts in migration behaviour. This study represents the first review of AIV surveillance data in Ireland to date. The findings highlight evolving patterns in virus distribution, seasonality and host dynamics, with implications for national surveillance strategies. Continued cross-species monitoring and integration of ecological data are essential to inform effective management strategies.

## Introduction

Avian influenza virus (AIV) is a global threat to both human and animal health, with some strains associated with high levels of morbidity and mortality [1]. Belonging to the *Orthomyxoviridae* family, these viruses are classified as influenza A, and subtyped based on two major antigens, haemagglutinin (H) and neuraminidase (N) [2]. These viruses are further categorised by their pathogenicity; low pathogenicity avian influenza (LPAI) and highly pathogenic avian influenza (HPAI), based on their virulence and impact on poultry [European Food Safety Authority (EFSA), 2025]. While LPAI typically causes milder clinical signs, the H5N1 virus is responsible for HPAI outbreaks belonging to the Goose/Guangdong lineage (Gs/Gd) [3].

Wild avifauna, particularly waterfowl and other water-associated birds, are recognized as key reservoirs of both pathotypes [4]. These species, alongside migratory birds, play a central role in the global dissemination of AIV through seasonal migrations, often disseminating the virus across continents [5]. In recent years, spillover events have involved mammals, such as mesocarnivores (foxes, seals) and, more recently, livestock, raising concerns about the expanding host range and potential for zoonotic transmission [6]. With the recent outbreak of HPAI H5N1 HPAI 2.3.4.4b in American cattle, cases have been reported in humans exposed to these animals, most commonly workers on farms [7].

In 2022 alone, HPAI outbreaks led to the culling of an estimated 50 million birds in affected European countries [8], causing not only extensive mortality but also disruption to poultry production and economic losses. The increasing prevalence and geographical spread of such events [9] underscores the need for not only enhanced surveillance but also an increased understanding of the various factors that are leading to outbreaks.

Ireland is a nation located in Northwestern Europe and is divided into four provinces: Leinster, Munster, Connacht and Ulster, with 26 counties forming the Republic of Ireland. As of its most recent population census, Ireland is home to over 5 million people [10], making it one of the smaller nations in terms of population within the European Union. Agriculture is a vital sector of the Irish economy, alongside services and technology. The poultry industry in Ireland, while smaller than other agricultural sectors, plays an important role. It is estimated that a significant proportion of poultry farming is commercial, with intensive operations concentrated in counties like Monaghan, Cavan and Cork [11]. The rest of the poultry population is raised in semi-commercial or backyard systems, which are more prominent in rural areas across the country. Backyard systems are generally small-scale and often integrate poultry with other forms of subsistence farming, relying on both traditional and improved breeds to meet household needs. Furthermore, Ireland plays a niche role as a major flyway for migratory birds, alongside its mild climate and abundance of wetlands [12].

Passive surveillance of AIV in wild birds in Ireland is implemented by the Department of Agriculture, Food and the Marine (DAFM), in accordance with European Commission Delegated Regulation 2020/689 [13]. This programme has been operational in Ireland since 2002, with periodic adaptations to optimise detection and resource allocation as necessary. Reports of sick or dead birds can be submitted to DAFM year-round through the Avian Check App or through local Regional Veterinary Offices [14]. Surveillance has been guided by a European Union (EU) - recommended target list of wild bird species for H5 HPAI detection [15], with additional investigations undertaken in the event of unusual mortality (>5 individuals of any species). This adaptive surveillance approach has underpinned Ireland’s ability to monitor and respond to HPAI in wild birds nationally.

Despite extensive global research on AIV, several key gaps in our knowledge remain. In Ireland, there is a lack of longitudinal analysis of AIV surveillance, limiting understanding of spatial, temporal and potential host-specific trends. This study aims to address this by conducting a desk-based review and analysis of AIV detections in the Republic of Ireland. This research aims to characterise the temporal, geographical and host-based patterns of AIV occurrence and assess the diversity and distribution of viral subtypes. The findings are intended to inform future surveillance priorities, guide national policy and support targeted research efforts, contributing to more effective disease monitoring and control strategies within the Irish context.

## Methods

### Data collection

A comprehensive literature search was conducted to collect data on AIV detections in the Republic of Ireland from the earliest reported case up to December 2024. The search included both peer-reviewed and grey literature sources to ensure completeness and accuracy. Primary sources consisted of official reports and databases from the DAFM, the Irish National Reference Laboratory for Virology (DAFM subdivision), the International System for Agricultural Science and Technology (AGRIS), the World Animal Health Information System (WAHIS) and the EFSA. Additionally, academic databases (PubMed, Scopus, Web of Science, CABI and Embase) were queried using search terms including ‘avian influenza’ AND ‘Ireland’.

Retrospective molecular data from PCR testing were included. To ensure a comprehensive temporal analysis of incidence, no start date for data collection was applied, with any data from early detections up to December 2024 included. Due to a lack of historical data on the number of samples/animals tested per year, only animals from which samples tested positive by molecular methods were included.

### Data extraction and curation

Data were imported into the web-based platform Google Sheets, with outbreaks recorded as individual rows in the dataset. All cases were georeferenced to the county level and included associated metadata when available. In the case of commercial farms, approximate coordinates were used to ensure privacy and protect farm locations. Domestic animal cases (i.e. poultry livestock) were aggregated as single entries due to the absence of specific animal counts, as per national guidelines on infection management. Due to insufficient data regarding the exact number of domestic animals affected during an outbreak, arising from national guidelines regarding clearing of infection, it was not possible to identify exact incidence in terms of population numbers. The dataset included key variables such as wild bird species, date of detection, county, geo coordinates (if available), influenza A RNA detection status and subtype. Wild bird species were taxonomically classified and grouped into categories (i.e. passerine, waterfowl, seabird, wader, raptor) to facilitate ecological analysis. Pathogenicity was classified as reported in surveillance records. For research papers, pathogenicity was determined based on the description provided by authors. Most records of AIV were obtained from public records and surveillance summaries. As these sources did not include genetic or lineage information, it was not possible to determine whether specific detections were associated with certain lineages (i.e. the Gs/Gd lineage), and therefore such information was not presented in the results.

Duplicate entries were removed, and records lacking confirmed influenza A RNA detection were excluded. Records with partial data (e.g. missing date or county) were retained when sufficient metadata allowed for spatial or temporal analysis. To ensure data accuracy, all entries were cross-referenced with multiple sources when available. In cases of data inconsistencies (e.g. differing reports on date of occurrence), priority was given to official sources [i.e. data arising from the National Reference Laboratory for Virology (DAFM)].

Temporal aggregation was performed at annual, monthly and seasonal levels to detect patterns. Migratory status was assigned using avian profiles specific to the Republic of Ireland, based on publicly available sources such as BirdWatch Ireland (https:// birdwatchireland.ie/category/migration/), the National Parks and Wildlife Services (https://www.npws.ie/researchprojects/ animalspecies/birds) and the National Biodiversity Data Centre (https://biodiversityireland.ie/birds/). The final curated dataset is available in Supplementary Material 1.

### Statistical and spatial analysis

Descriptive statistics were applied to analyse the overall dataset to include frequency distributions across years, counties, subtypes and host categories. Chi-square tests were utilised to assess seasonal variations in detections. To assess species-specific risk, z-scores, relative risk (RR) and 95% confidence intervals were calculated to identify species at a higher risk of AIV detection in relation to the overall dataset. Pearson’s correlation analysis was conducted to explore potential associations between AIV detections in wild birds and domestic poultry.

Linear regression analysis was utilised to examine trends in occurrence over time. Data analysis was carried out using Google Sheets and R Studio (2024, Version 12.1), while initial data cleaning and basic descriptive analysis were performed in Google Sheets. Statistical significance was set at *P*<0.05. Visualisations, including time series plots and geographical maps, were generated in R Studio to illustrate trends and spatial distributions. ArcGIS Pro (Version 3.2.0) was utilised to perform kernel density estimations and the creation of geographical maps.

## Results

### Descriptive characteristics of dataset

A comprehensive review of the literature identified 2,928 records of AIV in the Republic of Ireland between 1983 and 2024. Of these, 2,897 were fully subtyped, three cases were identified only to the H type, and 31 cases remained untyped (Table 1). The majority of cases involved avian wildlife (*n*=2,889; 98.7%), while domestic birds comprised a small portion (*n*=37; 1.3%). Two cases (0.07%) involved non-avian wildlife, both in red foxes (*Vulpes vulpes*), representing the only mammalian detections in the dataset. AIV records were documented in 25 of the 26 counties in Ireland, with no records identified for Carlow and 11 occurrences without county data.

**Table 1.** Summary of descriptive characteristics of AIV detections in Ireland (1983–2024)

Variable	Description	Wild bird	Domestic bird including poultry	Mammals	HPAI	LPAI	Unknown pathogenicity
		*n* (%)	*n* (%)	*n* (%)			
Totals	Totals	2,889	37	2	2,841	56	31
Year period	1983–1989	1 (0.03)	2 (5.41)		2 (0.07)	1 (1.79)	
	1990–1996	1 (0.03)	2 (5.41)			3 (5.56)	
	1997–2003	4 (0.14)			1 (0.04)	1 (1.79)	
	2004–2010	92 (3.18)	3 (8.11)		45 (1.58)		12 (38.71)
	2011–2017	1,151 (39.84)	1 (2.70)		1,147 (40.37)		12 (38.71)
	2018–2024	1,640 (56.77)	29 (78.38)	2 (100)	1,653 (58.18)	16 (28.57)	2 (16.12)
Subtype	H2N3	3 (0.10)				3 (5.56)	
	H5	3 (0.10)			1 (0.04)	2 (3.57)	
	H5N1	2,780 (96.22)	11 (29.73)	2 (100)	2,793 (98.31)		
	H5N2^*^		1 (2.70)			1 (1.79)	
	H3N8^*^		1 (2.70)			1 (1.79)	
	H5N6	3 (0.10)			3 (0.11)		
	H5N8	42 (1.45)	2 (5.41)		44 (1.55)		
	H6N1		14 (38.84)			14 (25)	
	H6N2	12 (0.42)				12 (21.43)	
	H6N5	1 (0.03)				1 (1.79)	
	H6N6	1 (0.03)				1 (1.79)	
	H6N8		1 (2.70)			1 (1.79)	
	H7N7		3 (8.11)			3 (5.56)	
	H9N2	1 (0.03)				1 (1.79)	
	H9N3	1 (0.03)				1 (1.79)	
	H10N2	1 (0.03)				1 (1.79)	
	H10N5	1 (0.03)				1 (1.79)	
	H10N6	1 (0.03)				1 (1.79)	
	H10N7	10 (0.35				10 (17.86)	
	H11N9	3 (0.10)				3 (5.56)	
	IAV	26 (0.90)	5 (13.51)				31 (100)
Season	Spring	564 (19.52)	13 (35.14)	1 (50)	564 (19.85)	10 (17.86)	4 (12.90)
	Summer	1,027 (35.55)	7 (18.92)		1,026 (36.11)	6 (10.71)	2 (16.12)
	Autumn	733 (25.37)	7 (18.92)	1 (50)	737 (25.84)		4 (12.90)
	Winter	562 (19.45)	6 (16.22)		512 (18.02)	35 (62.5)	21 (67.74)
	Unknown	3 (0.10)	4 (10.81)		2 (007)		5 (16.3)

*Detected simultaneously in the same flock of animals.

IAV, Influenza A virus.

In terms of subtype dynamics, H5N8 HPAI was the first subtype identified in Ireland; however, detailed location data were not available for this detection [16]. This was followed by H7N7 LPAI and H9N3, although detections were limited. The H5N1 HPAI subtype was the most prevalent, accounting for 2,793 cases (95.4%), and was the only subtype detected continuously between 2003 and 2024. H5N6 HPAI (*n*=3) was first detected in 2018, and H5N8 HPAI (*n*=44) was observed intermittently, with detections in 1983, 2016, 2017, 2020 and 2021. H5N6 HPAI and H5N3 were each only observed in isolated years. Of all detections, 2,841 (97%) were classified as HPAI, and 56 (1.9%) as LPAI. Subtypes H5N1, H5N8 and H5N6 were exclusively detected as HPAI, while H2N2, H2N3, H3N8, H6N1, H6N2, H6N5, H6N6, H7N7, H9N2, H9N3, H10N2, H10N5, H10N6, H10N7 and H11N9 were only observed as LPAI. Two H5 subtypes with undetermined N types were classified as LPAI, with one HPAI.

As shown in Supplementary Information 1, the earliest case of AIV detection reported by WAHIS was from 2016 (HPAI H5N8), whereas DAFM records indicate detections as early as 2003 (H5N1 HPAI); all cases prior to that were from academic publications.

### Temporal distribution of detections

The earliest detection of AIV in domestic avians occurred in 1983, involving H5N8 HPAI in a turkey flock [16]. Subsequent cases appeared sporadically; once in 1989 (H7N7), 1993 (H9N3) and 1997 (H9N2), and twice in 1995 (H7N7). However, detailed information from these outbreaks, such as county or other contextual data, was not available. No further cases were reported in domestic species until a single outbreak in 2007, followed by two outbreaks in 2009. A total of 37 cases were identified from 1983 to 2024, encompassing various domestic bird types, including laying hens (*n*=12), turkeys (*n*=11), ducks (*n*=5), poultry without a clear production line described (*n*=5) and non-poultry birds (*n*=4).

In wild birds, the earliest cases were recorded in 1983 and 1993, during which the H5N8 HPAI and H9N3 subtypes were identified in a duck of unrecorded species (*Anas* spp.) and a mallard (*Anas platyrhynchos*), respectively. In 2003, H5N1 HPAI was detected in a Mew Gull (*Larus brachyrhynchus*) in County Galway. Following these initial detections, no further HPAI cases were reported until 2005, when seven additional H5N1 HPAI cases were confirmed across multiple avian species. Between 2006 and 2009, annual cases remained low, ranging from two to ten cases per annum, until 2010, when an increase to 19 cases was noted.

A substantial increase in AIV detections occurred in subsequent years, with cases ranging from 21 to 47 until a pronounced increase to 219 cases occurred in 2015. The highest annual number of cases was between 2015 and 2019 (*n*=1,885; 64.38% of total), with the highest count in 2019 (*n*=874; 29.85% of total).

**Fig. 1.**
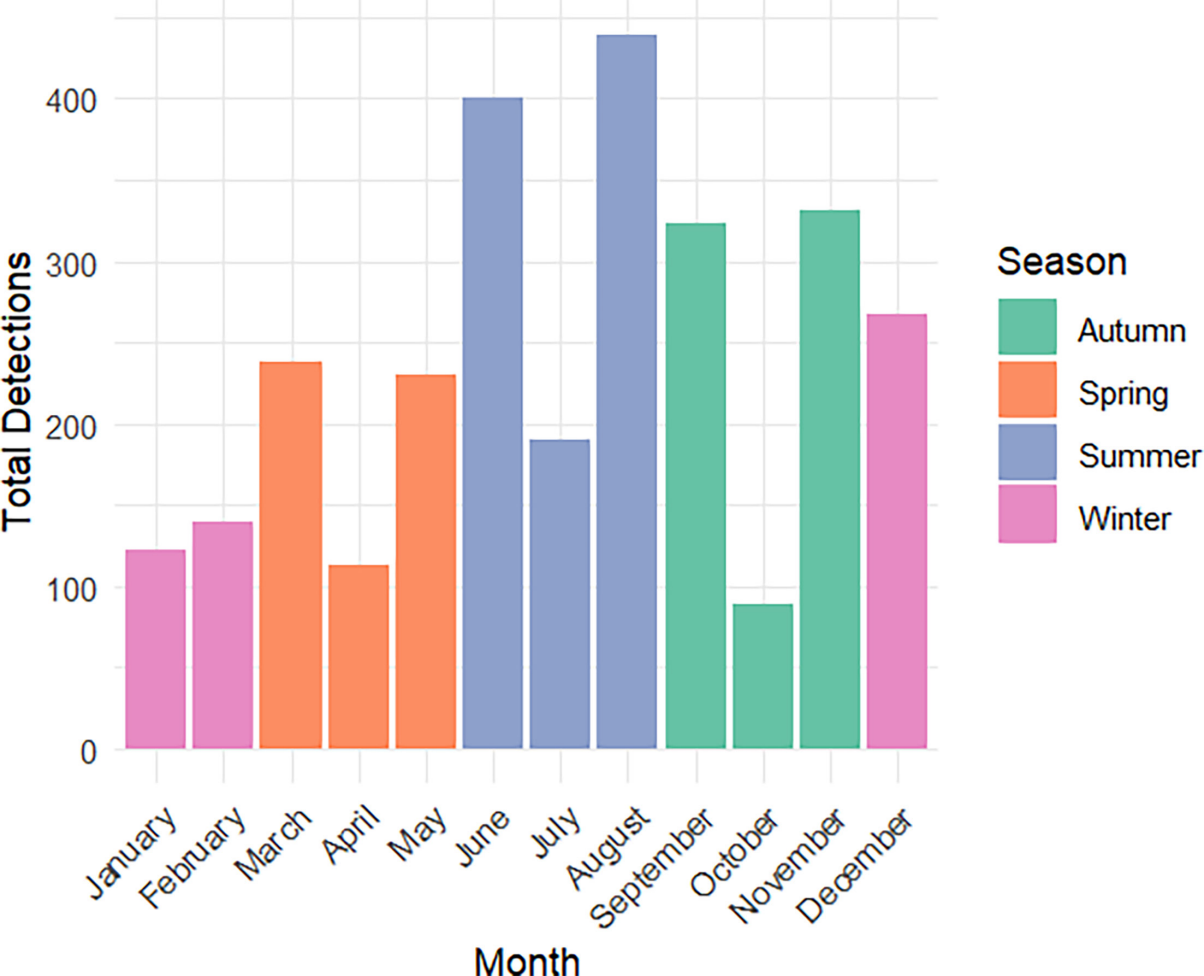
Monthly totals of AIV detections in Ireland, 1983–2024. Bars are coloured based on season.

Traditionally, influenza cases are believed to be associated with winter and early spring. To examine whether this pattern existed in Ireland, seasonality was assessed using a chi-square test. A total of seven cases lacked seasonal classification and were excluded from this analysis, with 2,921 cases included. Seasonal patterns indicated the highest detections in August (*n*=439; 15.03%), with summer being the most prevalent season overall (*n*=1,034; 35.4%), as seen in Fig. 1. The chi-square test confirmed significant variation in detections across seasons (χ^2^=194.3; *df=*3; *P*<0.05), with summer making up the highest individual chi-square value (χ^2^=126.35). The dataset was characterised by temporal clustering of cases, with 82.07% of cases occurring between 2015 and 2019.

Furthermore, all seasons had significant day-to-day variability in detections. Autumn had the highest median daily detection rate (3.82; range: 2–17), followed closely by summer (3.59; range: 2–31), as illustrated in Fig. S1 (available in the online Supplementary Material), indicating seasonal concentration and within-season fluctuation in AIV detections.

### Geographical distribution of cases

**Fig. 2.**
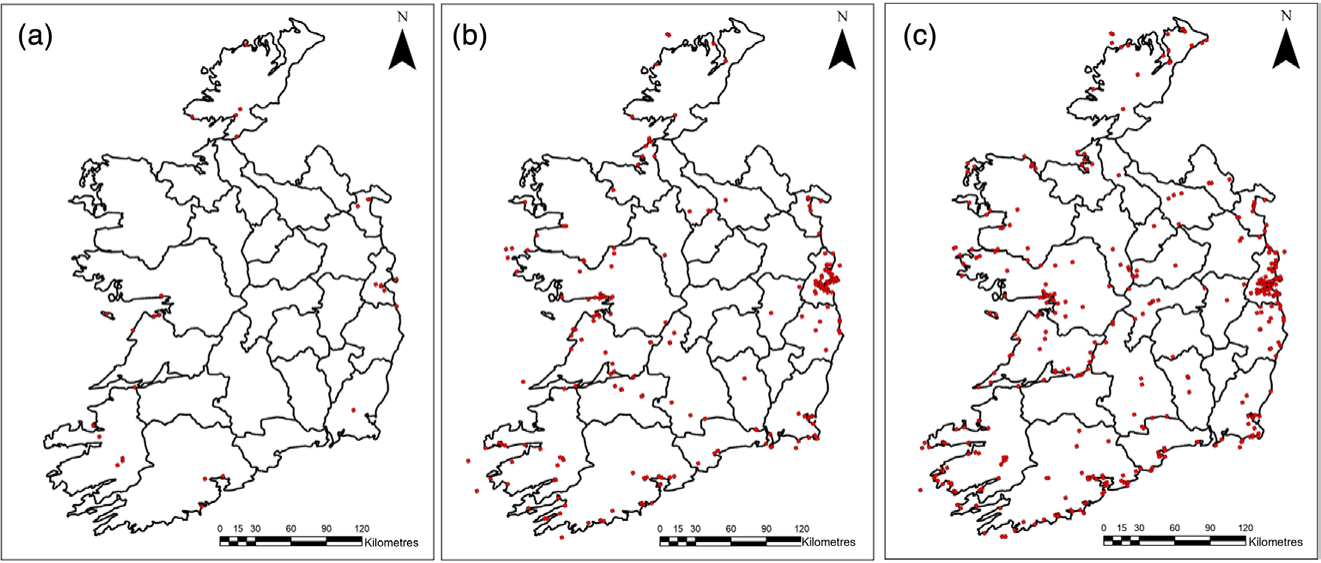
Spatiotemporal distribution of AIV detections in wild birds in the Republic of Ireland: (**a**) 2003–2010; (**b**) 2011–2017 and (**c**) 2018–2024.

Geospatial analysis identified County Dublin as the county with the highest AIV detections (*n*=1,208; 41.26%), followed by Galway (*n*=411; 14.04%) and Cork (*n*=212; 7.24%). Data prior to 2003 lacked location details and were therefore excluded. Notable geographical and temporal expansion was observed over the years. As illustrated in Fig. 2, early detections of AIV (2003–2010) in wild birds were relatively sparse and largely confined to coastal regions. During the years that followed (2011–2017), detections became more widespread, with emerging clusters in eastern counties, such as Dublin and surrounding areas. In the most recent years (2018–2024), AIV detections were reported across nearly all counties, with a marked increase in density in the east, alongside substantial clustering in parts of the midlands and west.

Distribution of the dominant subtype, H5N1 HPAI, was variable across the counties, reflecting its broad geographical spread. In contrast, other subtypes were more spatially restricted. H5N2 was detected in Wicklow and was detected in combination with H3N8 in a domestic flock. H5N8 HPAI was detected in Louth (*n*=2), while H5N6 HPAI was detected in both Tipperary (*n*=2) and Clare (*n*=1). H5N8 HPAI demonstrated the largest spatial range among non-H5N1 subtypes, with detections in 14 counties, namely Cork (*n*=10), Galway (*n*=5) and Monaghan (*n*=4), with two cases occurring in unknown counties.

**Fig. 3.**
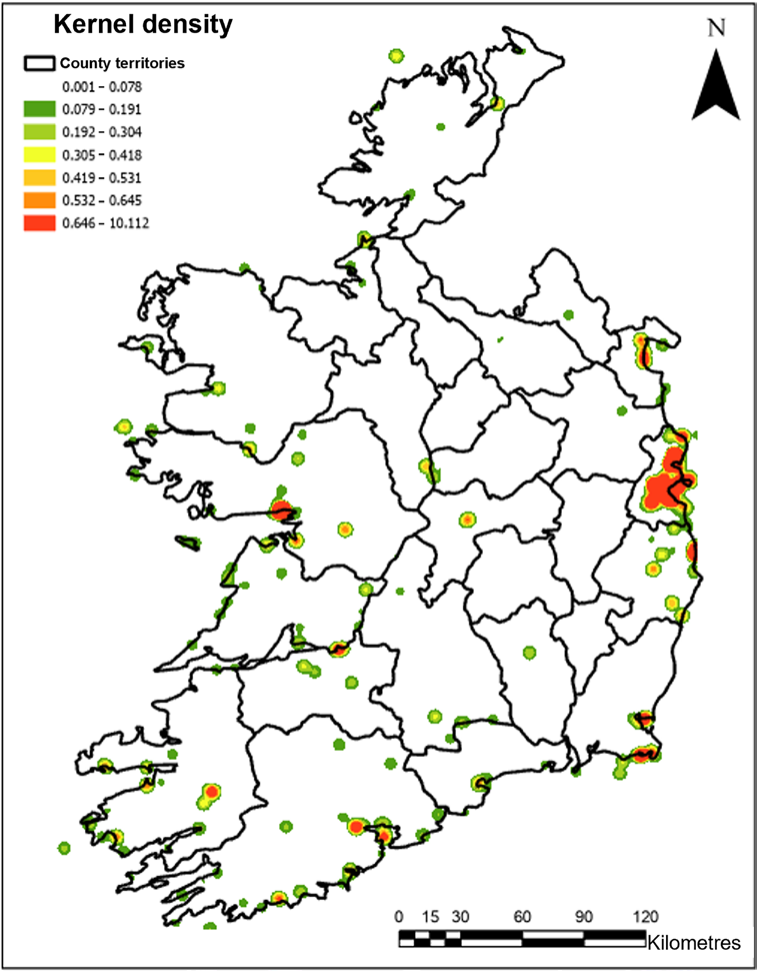
Kernel density estimation of the geographical distribution of AIV detections in the Republic of Ireland from 2003 to 2024. Density was visualized using sd contours, with intervals set at 1 sd. A red-to-green colour scale was used to indicate relative density, with red representing areas of higher detection concentration and green indicating lower density.

A kernel density estimation was performed on avifauna data using ArcGIS Pro to visualise the spatial distribution of AIV across Ireland from 2003 to 2024. As seen in Fig. 3, spatial heterogeneity in the distribution of AIV detections was observed. Density values ranged from 0.001 to 10.112 cases per square kilometre, with the highest densities seen in Dublin. Most clusters of density occurred in coastal regions, such as Cork and Galway. Lower density occurrences were dispersed throughout midland and western counties.

**Fig. 4.**
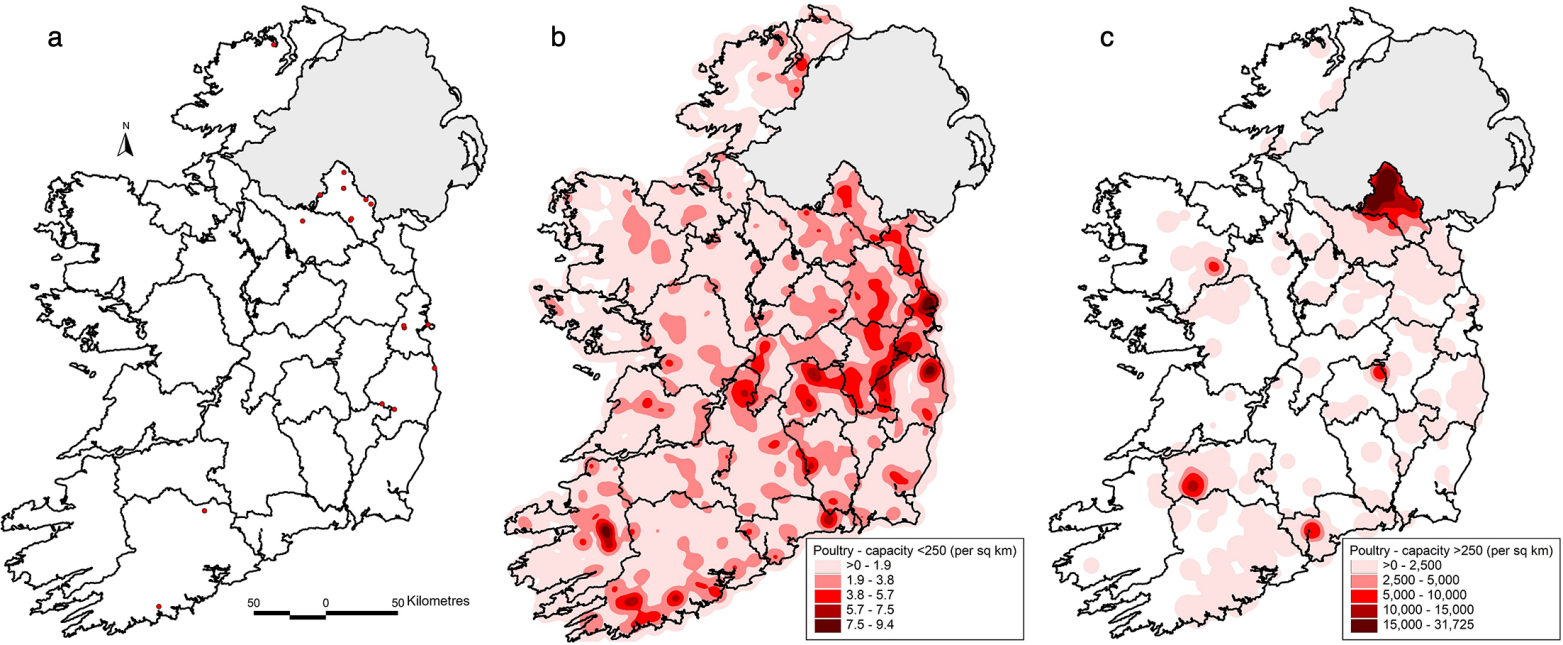
Spatial distribution of (a) AIV detections in domestic birds in the ROI (2003–2024); (b) density of small poultry holdings (i.e. less than 250 birds) and (c) density of large poultry holdings (i.e. more than 250 birds).

In contrast, the density of detections in domestic birds was more geographically confined, occurring primarily in counties with established poultry production, as seen in (Fig. 4). Notably, Monaghan emerged as a key hotspot, consistent with intensive commercial poultry operations. Additional outbreaks were identified in Wicklow, Cavan, Tipperary and Kerry, as seen in Fig. 4(a).

### Host-based patterns

Overall, 39 avian genera were identified in the dataset, with 28 entries unable to be categorised to a certain genus (namely domestic avians with no species listed). The most frequently affected genera were *Cygnus* spp. (*n*=454; 15.51%), *Chroicocephalus* spp. (*n*=340; 11.61%), *Ardea* spp. (*n*=261; 8.91%), *Larus* spp. (*n*=231; 7.89%) and *Egretta* spp. (*n*=227; 7.75%). Among these, *Cygnus* spp. had the most overall detections, with three different subtypes detected: H5N1 HPAI (*n*=416), H5N8 HPAI (*n*=32) and H5 HPAI (*n*=1). To identify avian genera with an elevated likelihood of AIV detection, z-scores were calculated based on the distribution of RR values across genera. *Cygnus* showed an elevated risk, with an RR of 5.97 and a z-score of 3.5, indicating a higher likelihood of detection relative to other genera. *Chroicocephalus* also exhibited an increased risk (RR=4.47; z=2.44), followed by *Ardea* (RR=8.5; z=1.56). While all three genera showed significantly elevated RR, they also accounted for a substantial proportion of overall detections. Beyond these, subsequent genera demonstrated a gradual decline in detection frequency and RR.

At a species level, the black-headed gull (*Chroicocephalus ridibundus*) emerged as the most frequently documented species (*n*=340; 11.61%). *Cygnus olor* (Mute Swan) represented the second most prevalent species (*n*=300; 10.24%). This was followed by *Ardea cinerea* (Grey Heron) with 261 observations (8.91%). Subsequent species showed gradual declines in frequency while maintaining statistically significant deviations from the mean, e.g. Little Egret (*n*=227; 7.75%). Eleven species had over 100 cases of AIV, whereas 23 species had less than five detections.

The majority of detections (*n*=1,339; 45.73%) occurred in waterfowl, followed by seabirds (*n*=657; 22.44%), waders (*n*=492; 16.80%) and raptors (*n*=244; 8.33%). Migratory species accounted for the majority of detections (*n*=1,575), with species considered partially migratory accounting for 298 detections. The first detection of AIV in wild birds in 1983 occurred in a partially migratory species; however, in the subsequent bout of detections, which occurred between 1993 and 2003, four out of five detections were in non-migratory species. Since then, with the exception of 2004–2010, detections were higher in migratory species compared to non-migratory species.

A Pearson’s correlation analysis was conducted using monthly aggregated data to assess the potential relationship between AIV in wild avifauna and domestic birds. For the Republic of Ireland, the correlation coefficient was −0.75 (*P*=0.7167), indicating no statistically significant linear relationship. Focusing on counties with a high density of commercial poultry farms (Cavan, Monaghan, Cork and Limerick) (Table 2), a statistically significant negative temporal correlation was observed (r=−0.279; *P*=0.031), suggesting that months with higher AIV detections in wild birds do not coincide with increased detection in domestic birds.

**Table 2.** Breakdown of case numbers per county with high density of commercial poultry farms (1983–2024)

County	Wild bird	Domestic flock
Cavan	7	1
Monaghan	5	21
Cork	209	2
Limerick	58	0

### Subtype distribution

Between 1983 and 2024, 19 AIV subtypes were detected in Ireland, with 31 records untyped and three typed as H5Nx. First recorded in 2003, H5N1 HPAI was the most prevalent (*n*=2,793), followed by H5N8 HPAI (*n*=44). In contrast, H6N1 was detected 14 times, whilst H5N6 was detected three times, both occurring in singular, separate years. Considerable variability in subtype distribution was observed across the years, with a median of 73.2 cases per year overall (Table 3).

**Table 3.** Total detections per subtype and median cases detected per year (over 40 years)

Subtype	Total detection	Median cases per year
H2N3	3	0.075
H3N8*	1	0.025
H5	3	0.075
H5N1	2,793	68.825
H5N2*	1	0.025
H5N6	3	0.075
H5N8	44	1.1
H6N1	14	0.35
H6N2	12	0.3
H6N5	1	0.025
H6N6	1	0.025
H6N8	1	0.025
H7N7	3	0.075
H9N2	1	0.025
H9N3	1	0.025
H10N2	1	0.025
H10N5	1	0.025
H10N6	1	0.025
H10N7	10	0.25
H11N9	3	0.075
IAV	31	0.78
Total	2,928	73.2

* Detected simultaneously in a flock

IAV, Influenza A virus.

**Fig. 5.**
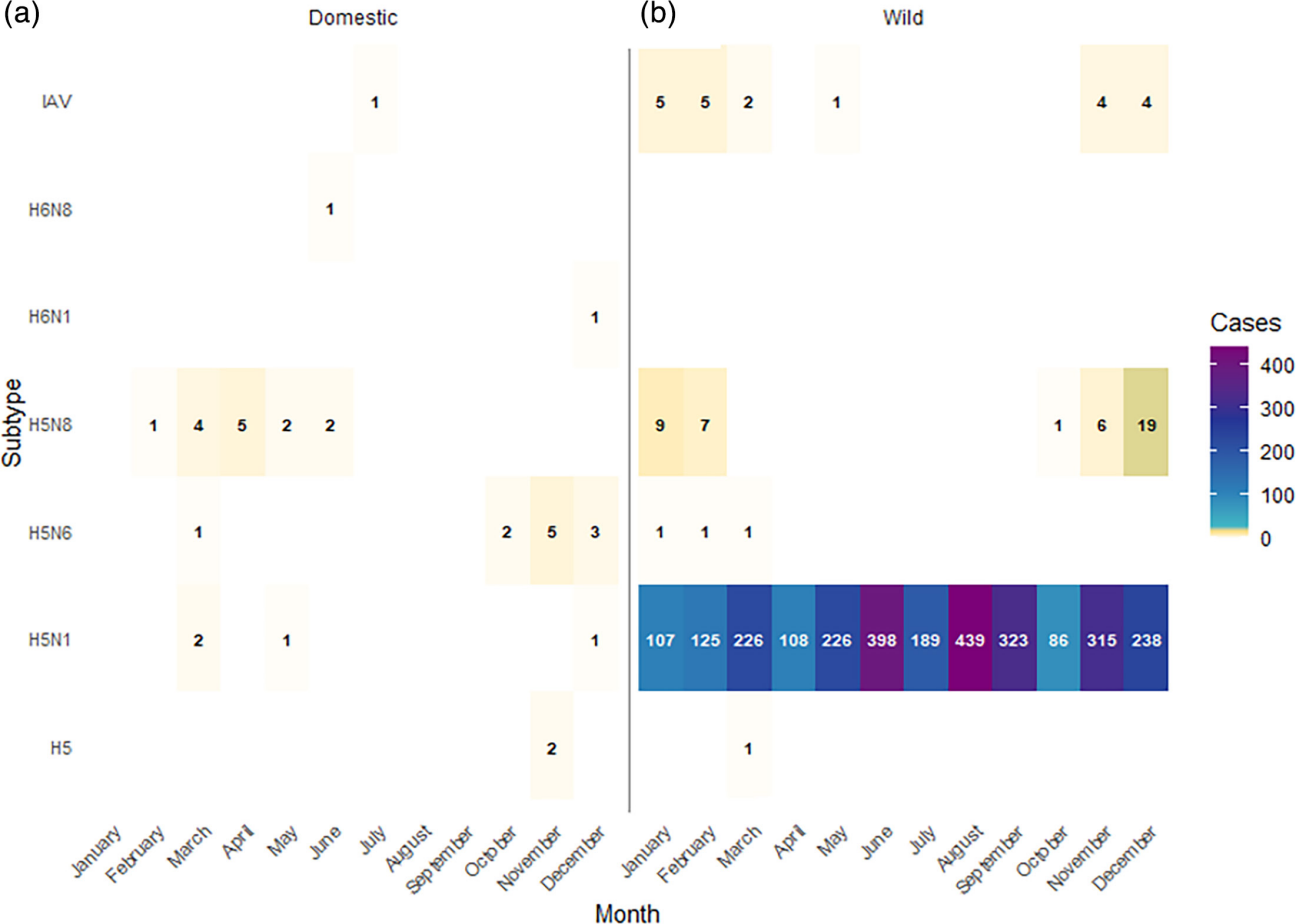
Seasonal distribution heatmap of (a) AIV detections by subtype in domestic species and (b) overall AIV detections by subtype. Subtypes H5N1, H5N6 and H5N8 are classified as HPAI, while H6N1 and H6N8 are classified as LPAI.

H5N1 HPAI occurred year-round, consistent with its presence during the study period. By contrast, H5N6 HPAI had singular detections in January, February and March. H5N8 HPAI had a strong seasonal pattern, with the highest frequency in December (*n*=20), followed by January (*n*=9), February (*n*=7), November (*n*=7) and October (*n*=1) as seen in Fig. 5(a). As seen in Fig. 5(b), the majority of these cases involved domestic species, with two non-domestic species affected in November.

Among domestic bird cases, H5N8 HPAI and H5N1 HPAI were the most frequently detected subtypes. H6N1 LPAI was also prevalent, particularly in laying hen flocks in Monaghan during 2020. The co-occurrence of multiple subtypes was observed in a duck game flock in Wicklow in 2021 (H5N2 LPAI and H3N8).

The year 2021 demonstrated the greatest diversity in terms of subtype detection, with concurrent detections of H5N1 HPAI, H5N8 HPAI and H6N8, and a co-occurrence of H5N2 and H3N8 in a duck flock. To assess whether the frequency of detections had increased over time, a linear regression analysis was performed. Results showed a slight positive increase in H5N1 HPAI over time; however, this was not statistically significant (*P*=0.153). Considering overall detections and subtypes, the regression model revealed a modest but statistically significant upward trend (*P*=0.044), with an estimated 7.6 detections per year.

## Discussion

This research presents a comprehensive analysis of 2,888 records of AIV detections in the Republic of Ireland over a 41-year period. The findings of this study are consistent with global patterns, identifying wild avifauna as reservoirs of AIV [17,18]. A notable finding of this research is the seasonal distribution of detections, which peaked during the summer months, in contrast to the common viewpoint that AIV is predominantly a winter-associated pathogen [5,19]. The substantial increase in reported detections, particularly between 2015 and 2019, may be indicative of either a rise in prevalence or an increase in national surveillance efforts. Given the significant seasonal variation observed (particularly the dominance of summer in the overall dataset and autumn in the sensitivity analysis), this divergence highlights the need for further investigation into potential ecological drivers, virus genetics, such as variations in pathogenicity, and host behaviours that could influence virus transmission.

Since 2005, outbreaks of H5N1 HPAI in Europe have been associated with the Gs/Gd lineage viruses and subsequent clades [20]. This lineage has been responsible for repeated outbreaks globally, with migratory waterbirds implicated in long-distance travel of the virus from Asia to Europe [21]. More recently, Bellido-Martín *et al.* [22] highlighted how the evolution and transmission of H5 viruses within the Gs/Gd lineage have changed their epidemiology in Europe, including increased involvement of wild bird populations and greater persistence across seasons. The prominent role of waterbirds was evident in the detections collated in this study. In the Irish context, several factors may contribute to the epidemiology of these viruses. Ireland’s location on the migratory bird path has resulted in large numbers of overwintering waterfowl species, such as swans, geese and ducks [12]. These species congregate in coastal and inland waterbodies, creating opportunities for viral introduction and dissemination. The relatively mild oceanic climate, enhanced in recent years by climate change, may potentially prolong the period of congregation [23], as seen in detections in migratory birds outside of the standard periods. Although the timing and geographical context of reported detections in Ireland suggest involvement of the Gs/Gd lineage, sequence data were not available for genetic confirmation, and therefore, exact lineage attribution could not be established. As more sequence data become available, depositing sequences in publicly accessible databases is recommended to facilitate analyses of evolutionary history, lineage assignment and relationships among viruses.

Occasional detections of AIV in mammals may reflect environmental contamination or potentially scavenger-mediated transmission pathways. These observations highlight the need for further research into the role of non-avians in the prevalence of AIV [6,24]. To the best of the authors’ knowledge, this is the first study to systematically collate and analyse AIV records in Ireland, spanning over four decades of data.

Beyond surveillance-related explanations, it is essential to consider broader ecological and behavioural factors that could influence temporal increases in detection. For example, while increased surveillance [3] and fluctuations in migratory bird populations are likely contributors, long-term data indicate significant shifts in bird populations. As outlined in the recent Avian Check App Report (2025), reports of deceased or sick birds increased from 161 reports in 2021 to 2,516 in 2022. This correlates with 265 wild birds tested by passive surveillance in 2021 [25] and 202 in 2022 [25], increasing to 424 in 2023 [26]. A report issued in 2019 detailed that wintering waterbirds suffered a population decrease of 40% over the previous 17 years. However, some species, such as the whooper swan, saw increases in recent years, with a 13.4% change [27]. These alterations in host dynamics suggest the possibility of altered migratory pathways in Ireland, potentially influenced by climate change. For example, detections of AIV in birds outside their normal period of residency were observed in this research. Notably, the whooper swan (*n*=151), a species that mainly overwinters in Ireland from October to April, had 19 detections occurring outside of this period. Similarly, the Northern Pintail (*n*=40), a winter visitor, had two detections in June, while the greater scaup (*n*=37) had three recorded detections in May. The common eider (*n*=44), another wintering species, had ten detections outside of its expected seasonal range, as seen in Supplementary Material. These irregularities could reflect changes in migration patterns or shifts in overwintering timepoints, potentially driven by climate change [28], which may also be impacting virus dynamics, evolution and transmission [23].

Species-specific trends reinforce the importance of surveillance and research into certain hosts and host dynamics. Increases in detection over time in species such as swans (*Cygnus* spp.) align with existing literature, recognising them as a key reservoir of both low pathogenicity and highly pathogenic influenza [29,30]. The significantly increased z-scores for these birds indicate non-random patterns of infection, potentially driven by habitat-based exposures or possibly virus adaptation. Water-associated birds such as swans, gulls, herons and egrets are well-documented reservoirs of AIV due to their presence in aquatic environments [31,33]. Similar trends are observed across Europe in waterfowl and other aquatic birds, with recent research showing strong associations between waterbird community composition and HPAI H5 occurrences, suggesting that transmission processes are similar across regions and highlighting the importance of region-specific surveillance and risk assessment. Additional studies in Great Britain [34], France [35] and Serbia further highlight the role of waterfowl in HPAI transmission and the repeated introduction of H5N into Europe [36].

Passive surveillance of wild birds in Ireland is inherently influenced by targeted sampling strategies, which may lead to certain species being over- or underrepresented in the data. Surveillance in Ireland is conducted in line with DAFM protocols and the EU target list of birds for H5 HPAI. Current protocols stipulate collection and testing in the event of a discovery of any raptor, three or more waterbirds, or five or more of any additional species [37]. However, in the case of unprecedented mortality, representative sampling occurs to ensure epidemiological evaluation while maintaining sampling resources to respond elsewhere. Discrepancies in the earliest year of AIV detection reporting were observed across data sources, with WAHIS listing the first detection in 2016, DAFM records from 2003, and earlier detections (pre-2003) available only through academic publications. These differences likely reflect variation in archival practices, database coverage and changes in reporting practices, rather than absence of detections, and highlight the importance of integrating multiple sources to present a comprehensive national history of AIV detections.

Spatial clustering of AIV detections in Ireland appears to be influenced by a combination of anthropogenic and ecological factors, including population density, proximity to water bodies and distribution of targeted surveillance strategies. The country’s capital, Dublin, accounted for ~41% of reported detections, a disproportionately high figure. Multiple factors could be considered as drivers of this, including human population density. Results from the most recent Irish Census in 2022 revealed that Dublin accounted for ~40% of the total Irish population [38]. Furthermore, this increased population density could impact reporting. In January 2021, a virtual reporting platform was created by DAFM to facilitate the reporting of birds potentially affected by AIV. To date, 3,519 reports have been submitted via this platform (January 2021–March 2025), with the highest reports submitted from areas of dense human population and coastal counties [39]. Kernel density estimations confirmed these findings, with high-density clusters visualised in Dublin, Galway and Cork regions.

As seen in the Avian Check App report, a spike in reports was observed in September and October of 2022, coinciding with increased AIV cases in gannets globally [40]. As seen in the data presented in this research, of the 97 detections in 2022, 52 were detected in September and October, of which 48 were northern gannets. Similarly, in the data published by Giralt Paradell *et al*. [41], increased cases of HPAI were observed in gannets and an estimated minimum local population mortality of 3,126 birds (95% confidence intervals: 2,993–3,260). Comparable mortality events were observed in other European seabird colonies, with 710 detections in 2022 across Europe [25]. These detections included colony-breeding seabirds along the northwest coast of Europe, suggesting that the events observed in the Irish gannet populations were part of a European-wide outbreak, highlighting the need for coordinated monitoring and management strategies.

The multi-county occurrence of H5N1 HPAI reflects its well-documented virulence and ability to rapidly spread in a population [42,43]. Conversely, the limited spatial distribution of other subtypes, such as H5N3 and H5N6 HPAI, suggests the potential for isolated introductions or localised spillover events. Interestingly, H6N1 was only detected in domestic birds in 2020 and was restricted to the county of Monaghan. This spatial clustering, along with reported detections in Northern Ireland, suggests potential farm-to-farm transmission [44]. McMenamy e*t al*. [44] reported that the first detection in the Republic of Ireland occurred on a farm located 100 m away from a known positive premises in Northern Ireland. Although a definitive source could not be defined in this outbreak [44], a wild bird origin remains likely given the global prevalence in wild birds at that time [45,48]. However, to date, H6N1 has not been detected in wild birds in the Republic of Ireland, likely due to poultry being reared mostly inland and away from coastal wetlands, where waterfowl density would be increased. Furthermore, the virus can also be transmitted through the environment, particularly via contaminated water or surfaces, where influenza is known to potentially persist for extended periods [49]. In the case of H5N8 HPAI, this subtype was detected in a turkey flock in Wicklow in early December 2020, just after detection of the same subtype in a whooper swan just under 40 km away. The lack of a statistically significant correlation between wild avifauna and domestic AIV cases in this study suggests a potential delay in direct spillover in an Irish context. These findings highlight the importance of integrating spatial and ecological data to inform surveillance strategies and improve understanding of environmental and anthropogenic factors influencing viral distribution and persistence. Considering a poultry-dense location such as Monaghan, H5N1 HPAI was detected in domestic birds in November and December of 2021, with wild bird detections limited to a single corvid detection in December 2021. The timing and consistency of outbreaks in domestic flocks suggest that farm-to-farm transmission occurred, or that the virus was circulating undetected in the wild bird population. This is highlighted in Cork in 2019, where H5N1 HPAI was detected in May in multiple wild bird species, alongside an outbreak in a non-poultry flock. The detection across various species at the same time, and during months consistent with wild bird migration, suggests the initial introduction to poultry establishments occurs from wild birds, followed by local amplification through farm-to-farm transmission. To create risk-based models and effective biosecurity plans for farms, there is a clear need for continuation and expansion of intensive and targeted wild animal and environmental surveillance, alongside utilisation of temporal surveillance data to infer potential transmission routes.

The detection of AIV in red foxes is particularly concerning, as it underscores the potential for the virus to spill over into non-avian hosts. A recent Irish serological study conducted between 2022 and 2024 detected influenza A antibodies in mesocarnivore species; red foxes, Eurasian Badgers (*Meles meles*) and American Mink (*Neogale vison*). This study utilised a commercially available ELISA, screening 219 samples, of which 31 tested positive for influenza A antibodies. Of these, 23 of 28 antibody-positive fox samples were typed as H5, along with the singular badger sample. Two antibody-positive samples from American Mink could not be subtyped, testing negative for both H5 and H7 [50]. These findings are consistent with broader European surveillance efforts, where seroprevalence of various influenza A antibodies has been documented in multiple wild mammal species, including seals, foxes, martens, polecats, badgers and stoats [51,53]. The ability of these animals to survive infection and retain antibodies suggests wild mammals could act as incidental hosts or reservoirs of influenza A viruses, highlighting the importance of incorporating mammalian wildlife into AIV surveillance networks.

Alongside this, growing concerns for livestock have arisen, given the recent spillover of H5N1 HPAI 2.3.4.4b into dairy cattle across several states in the USA [54,55]. Moreover, the detection of H5N1 HPAI in a sheep in Yorkshire, UK [56], further highlights the expanding host range of the virus. To date, AIV has not been detected in livestock in Ireland; nevertheless, these events signify a concerning shift in the epidemiology of HPAI. The evolution of host dynamics highlights the need for enhanced cross-species and cross-sectoral surveillance, and the use of One Health approaches to monitor and mitigate potential risks.

The retrospective nature of this analysis, and reliance on existing surveillance records, introduces inherent biases, including variable reporting practices, surveillance intensity and sampling efforts. Poultry data, in particular, may be incomplete due to the need to protect farmers’ privacy. Additionally, changes in surveillance intensity over time may have skewed detection rates and temporal trends. Mammalian data were both limited in frequency and geographic scope; however, they highlight the need for inclusion of non-avian species in future surveillance frameworks. Kernel density estimation and z-score analyses provide visualization of spatial clustering and relative species-level detection, but do not formally assess whether observed patterns occur by chance. Future research should include analyses of spatial patterns and sequencing of detections to determine similarity and differences among occurrences. Despite these limitations, the findings of this analysis provide valuable insights into long-term trends and emerging risks associated with AIV in Ireland and potentially beyond.

## Conclusion

This study provides the most comprehensive longitudinal analysis of AIV in the Republic of Ireland to date, spanning four decades and including both avian and non-avian hosts. These findings highlight clear temporal and spatial trends, with urbanised clustering. Seasonal peaks during summer months – particularly between 2015 and 2019 – contrast with the normal belief that AIV is predominantly a winter-associated pathogen, suggesting the influence of shifting migratory behaviours or the impacts of climate change. Specific avian genera were more commonly affected, underscoring the importance of species-specific targeting in surveillance and monitoring efforts. Compared with other European countries, Ireland generally shows lower overall AIV incidence, although temporal patterns and implicated host species are broadly consistent with continental trends. This research makes important contributions to the evidence base for understanding the environmental-, host- and temporal-related factors impacting AIV occurrence in Ireland. Further research is needed to elucidate the potential for farm-level outbreaks, the persistence of AIV in wild bird populations throughout the year, and the potential role of mammals in the dissemination of the virus.

## Supplementary material

10.1099/jgv.0.002218 Uncited Fig. S1.

10.1099/jgv.0.002218 Supplementary Material 1.

## Abbreviations

AGRIS: Agricultural Science and Technology
AIV: avian influenza virus
DAFM: Department of Agriculture, Food and the Marine
EFSA: European Food Safety Authority
Gs/Gd: Goose/Guangdong lineage
HPAI: highly pathogenic avian influenza
LPAI: low pathogenicity avian influenza
RR: relative risk
WAHIS: World Animal Health Information System

## References

[1] Charostad J, Rezaei Zadeh Rukerd M, Mahmoudvand S, Bashash D, Hashemi SMA (2023). A comprehensive review of highly pathogenic avian influenza (HPAI) H5N1: an imminent threat at doorstep. Travel Med Infect Dis.

[2] Tan MJ (2012). Netter’s Infectious Diseases.

[3] Department of Agriculture, Food and the Marine (2024). Avian influenza surveillance programme. https://www.animalhealthsurveillance.agriculture.gov.ie/media/Avian%20Influenza.pdf.

[4] Bodewes R, Kuiken T (2018). Changing role of wild birds in the epidemiology of avian influenza A viruses. Adv Virus Res.

[5] Kandeil A, Patton C, Jones JC, Jeevan T, Harrington WN (2023). Rapid evolution of A(H5N1) influenza viruses after intercontinental spread to North America. Nat Commun.

[6] Peacock TP, Moncla L, Dudas G, VanInsberghe D, Sukhova K (2025). The global H5N1 influenza panzootic in mammals. Nature.

[7] Mostafa A, Naguib MM, Nogales A, Barre RS, Stewart JP (2024). Avian influenza A (H5N1) virus in dairy cattle: origin, evolution, and cross-species transmission. mBio.

[8] Adlhoch C, Fusaro A, Gonzales JL, Kuiken T, Marangon S (2023). Avian influenza overview September - December 2022. EFSA J.

[9] Parums DV (2023). Editorial: global surveillance of highly pathogenic avian influenza viruses in poultry, wild birds, and mammals to prevent a human influenza pandemic. *Med Sci Monit*.

[10] Central Statistics Office (2024). Population and migration estimates, April 2024. https://www.cso.ie/en/releasesandpublications/ep/p-pme/populationandmigrationestimatesapril2024/keyfindings/.

[11] Kelleghan DB, Hayes ET, Everard M, Curran TP (2020). Assessment of the impact of ammonia emissions from intensive agriculture installations on special areas of conservation and special protection areas.

[12] Crowe O, Wilson J, Aznar I, More SJ (2009). A review of Ireland’s waterbirds, with emphasis on wintering migrants and reference to H5N1 avian influenza. Ir Vet J.

[13] European Commision (2020). Commission delegated regulation (EU) 2020/689. https://www.efsa.europa.eu/en/topics/topic/avian-influenza.

[14] Department of Agriculture, Food and the Marine (2020). Avian influenza (bird flu). https://www.gov.ie/en/department-of-agriculture-food-and-the-marine/publications/avian-influenza-bird-flu/.

[15] Reinartz R, Slaterus R, Foppen R, Stahl J (2024). Update of the target list of wild bird species for passive surveillance of H5 HPAI viruses in the EU. *EFS3*.

[16] McNulty MS, Allan GM, McCracken RM, McParland PJ (1985). Isolation of a highly pathogenic influenza virus from turkeys. Avian Pathol.

[17] Ren P, Gao Z, Li X, Tang J, Li P (2024). Phylogeography and biological characterization of H12N2 virus isolated from whooper swan in Central China. Front Microbiol.

[18] Yang L, Fan M (2025). Reaction-advection-diffusion model of highly pathogenic avian influenza with behavior of migratory wild birds. J Math Biol.

[19] Tuncer N, Martcheva M (2013). Modeling seasonality in avian influenza H5N1. J Biol Syst.

[20] Verhagen JH, Fouchier RAM, Lewis N (2021). Highly pathogenic avian influenza viruses at the wild-domestic bird interface in Europe: future directions for research and surveillance. Viruses.

[21] Caliendo V, Lewis NS, Pohlmann A, Baillie SR, Banyard AC (2022). Transatlantic spread of highly pathogenic avian influenza H5N1 by wild birds from Europe to North America in 2021. Sci Rep.

[22] Bellido-Martín B, Rijnink WF, Iervolino M, Kuiken T, Richard M (2026). Evolution, spread and impact of highly pathogenic H5 avian influenza A viruses. Nat Rev Microbiol.

[23] Prosser DJ, Teitelbaum CS, Yin S, Hill NJ, Xiao X (2023). Climate change impacts on bird migration and highly pathogenic avian influenza. Nat Microbiol.

[24] Velkers FC, Blokhuis SJ, Veldhuis Kroeze EJB, Burt SA (2017). The role of rodents in avian influenza outbreaks in poultry farms: a review. Vet Q.

[25] Aznar I, Baldinelli F, Stoicescu A, Kohnle L, European Food Safety Authority (EFSA) (2022). Annual report on surveillance for avian influenza in poultry and wild birds in Member States of the European Union in 2021. *EFS2*.

[26] Abrahantes JC, Aznar I, Catalin I, Kohnle L, Mulligan KF (2025). Avian influenza annual report 2023. *EFS2*.

[27] Burke B, Lewis LJ, Fitzgerald N, Frost T, Austin G (2018). Estimates of waterbird numbers wintering in Ireland, 2011/12–2015/16. Irish Birds.

[28] Rubolini D, Saino N, Møller AP (2010). Migratory behaviour constrains the phenological response of birds to climate change. Clim Res.

[29] Bergervoet SA, Pritz-Verschuren SBE, Gonzales JL, Bossers A, Poen MJ (2019). Circulation of low pathogenic avian influenza (LPAI) viruses in wild birds and poultry in the Netherlands, 2006-2016. Sci Rep.

[30] Lambrecht B, Marché S, Houdart P, van den Berg T, Vangeluwe D (2016). impact of age, season, and flowing vs. stagnant water habitat on avian influenza prevalence in mute swan (*Cygnus olor*) in Belgium. Avian Dis.

[31] Sheikh MOB, Rashid PMA, Marouf AS, Rahim ZH, Saeed SS (2025). Molecular characterization and genetic analysis of highly pathogenic H5N1 clade 2.3.4.4b in seagulls from Dukan Lake, Iraq. Virus Genes.

[32] Soda K, Tomioka Y, Usui T, Uno Y, Nagai Y (2022). Susceptibility of herons (family: *Ardeidae*) to clade 2.3.2.1 H5N1 subtype high pathogenicity avian influenza virus. Avian Pathol.

[33] Woo C, Kwon J-H, Lee D-H, Kim Y, Lee K (2017). Novel reassortant clade 2.3.4.4 avian influenza A (H5N8) virus in a grey heron in South Korea in 2017. Arch Virol.

[34] Shemmings-Payne W, De Silva D, Warren CJ, Thomas S, Slomka MJ (2024). Repeatability and reproducibility of hunter-harvest sampling for avian influenza virus surveillance in Great Britain. Res Vet Sci.

[35] Briand F, Niqueux E, Schmitz A, Martenot C, Cherbonnel M (2022). Multiple independent introductions of highly pathogenic avian influenza H5 viruses during the 2020–2021 epizootic in France. Transbound Emerg Dis.

[36] Šolaja S, Glišić D, Veljović L, Milošević I, Nićković E (2025). Phylogeographic analysis of clade 2.3.4.4b H5N1 in Serbia reveals repeated introductions and spread across the Balkans. Pathogens.

[37] DAFM (2025). Avian influenza update: 8th April 2025. https://www.animalhealthsurveillance.agriculture.gov.ie/media/animalhealthsurveillance/Avian%20Influenza%20Update%20No%206%20of%202025%20.pdf.

[38] Central Statistics Office (2022). Press statement: Census of Population 2022 – summary results, Dublin. https://www.cso.ie/en/csolatestnews/pressreleases/2023pressreleases/pressstatementcensusofpopulation2022-summaryresultsdublin/.

[39] Department of Agriculture, Food and the Marine (2025). Avian check app report. https://assets.gov.ie/static/documents/Avian_Check_App_2021-2025_report_280425.pdf.

[40] Lane JV, Jeglinski JWE, Avery‐Gomm S, Ballstaedt E, Banyard AC (2024). High pathogenicity avian influenza (H5N1) in Northern Gannets (*Morus bassanus*): global spread, clinical signs and demographic consequences. Ibis.

[41] Giralt Paradell O, Goh T, Popov D, Rogan E, Jessopp M (2023). Estimated mortality of the highly pathogenic avian influenza pandemic on northern gannets (*Morus bassanus*) in southwest Ireland. Biol Lett.

[42] Banyard AC, Bennison A, Byrne AMP, Reid SM, Lynton-Jenkins JG (2024). Detection and spread of high pathogenicity avian influenza virus H5N1 in the Antarctic Region. Nat Commun.

[43] Mao Q, Li Z, Li Y, Zhang Y, Liu S (2024). H5N1 high pathogenicity avian influenza virus in migratory birds exhibiting low pathogenicity in mallards increases its risk of transmission and spread in poultry. Vet Microbiol.

[44] McMenamy MJ, McKenna R, Bailie VB, Cunningham B, Jeffers A (2024). Evaluating the impact of low-pathogenicity avian influenza H6N1 outbreaks in United Kingdom and Republic of Ireland poultry farms during 2020. Viruses.

[45] Everest H, Hill SC, Daines R, Sealy JE, James J (2020). The evolution, spread and global threat of H6Nx avian influenza viruses. Viruses.

[46] Muzyka D, Pantin-Jackwood M, Spackman E, Stegniy B, Rula O (2012). Avian influenza virus wild bird surveillance in the Azov and Black Sea regions of Ukraine (2010-2011). Avian Dis.

[47] Siembieda JL, Johnson CK, Cardona C, Anchell N, Dao N (2010). Influenza A viruses in wild birds of the Pacific flyway, 2005-2008. Vector Borne Zoonotic Dis.

[48] Trogu T, Bellini S, Canziani S, Carrera M, Chiapponi C (2024). Surveillance for avian influenza in wild birds in the Lombardy region (Italy) in the period 2022–2024. Viruses.

[49] Rohani P, Breban R, Stallknecht DE, Drake JM (2009). Environmental transmission of low pathogenicity avian influenza viruses and its implications for pathogen invasion. Proc Natl Acad Sci USA.

[50] Ruy P-E, Ball S, Barry G, Cuq B, McDevitt AD (2025). Expanding wildlife serosurveillance: a study of influenza A virus exposure in Irish carnivores. Eur J Wildl Res.

[51] Bodewes R, Rubio García A, Brasseur SM, Sanchez Conteras GJ, van de Bildt MWG (2015). Seroprevalence of antibodies against seal influenza A(H10N7) virus in harbor seals and gray seals from the Netherlands. PLoS One.

[52] Chestakova IV, van der Linden A, Bellido Martin B, Caliendo V, Vuong O (2023). High number of HPAI H5 virus infections and antibodies in wild carnivores in the Netherlands, 2020–2022. Emerg Microbe Infect.

[53] Gholipour H, Busquets N, Fernández-Aguilar X, Sánchez A, Ribas MP (2017). Influenza A virus surveillance in the invasive American mink (*Neovison vison*) from freshwater ecosystems, northern Spain. Zoonoses Public Health.

[54] Baker AL, Arruda B, Palmer MV, Boggiatto P, Sarlo Davila K (2025). Dairy cows inoculated with highly pathogenic avian influenza virus H5N1. Nature.

[55] Caserta LC, Frye EA, Butt SL, Laverack M, Nooruzzaman M (2024). Spillover of highly pathogenic avian influenza H5N1 virus to dairy cattle. Nature.

[56] Mahase E (2025). H5N1: UK reports world’s first case in a sheep. BMJ.

