# Supplementary material for: Avian influenza in Ireland: a spatiotemporal, subtype and host-based analysis (1983–2024)
Source: J Gen Virol. 2026 Feb 25;107(2):002218. doi: 10.1099/jgv.0.002218 (PMC12935314; doi:10.1099/jgv.0.002218)
Supplement: Uncited Fig. S1. [file jgv-107-02218-s001.pdf]

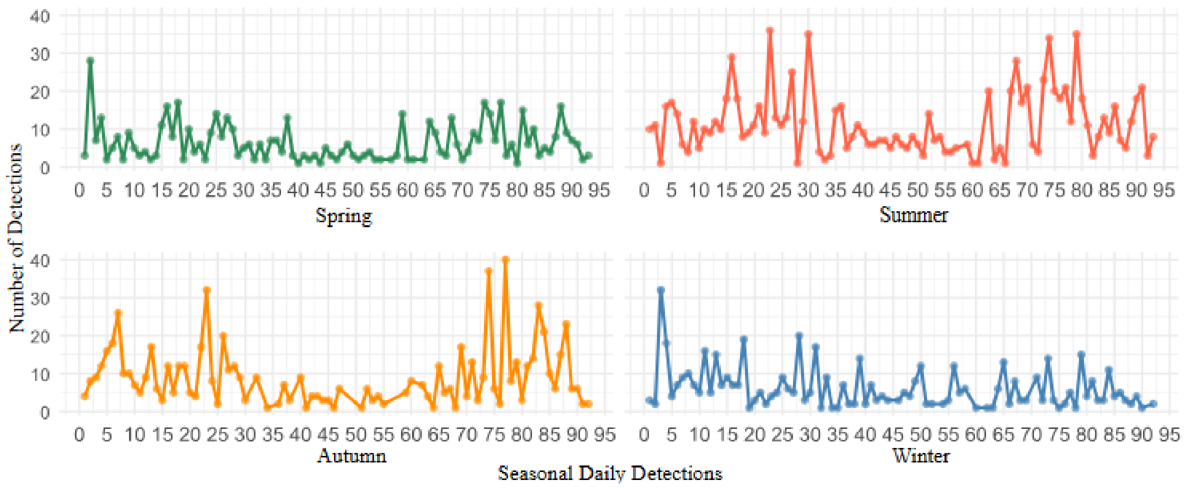

Fig. 1. Seasonal daily detections of avian influenza. Time series plot details spring- March, April, May (green), summer - June, July, August (red), autumn - September, October, November (yellow) and winter - December, January February (blue). The vertical axis details the number of detections per day, with the horizontal axis showing the days occurring in each season.
